# Mitochondria-Targeting microRNAs (mitomiRs): Potential Mediators of Environmental Mitoepigenetics in Mammalian Spermatogenesis

**DOI:** 10.3390/biom16060804

**Published:** 2026-05-29

**Authors:** Vanessa Zak, Jonathan LaMarre

**Affiliations:** Biomedical Sciences, University of Guelph, Guelph, ON N1G 2W1, Canada

**Keywords:** epigenetics, mitoepigenetics, male fertility, spermatogenesis, mitochondria, environmental stress, microRNA

## Abstract

The field of mitoepigenetics involves the investigation of modifications in mitochondrial DNA (mtDNA), genomic DNA that encodes mitochondrial proteins, and the expression of RNAs that regulate mitochondrial gene expression but do not alter the DNA sequence. This area of research is growing rapidly and has substantial relevance to male mammalian fertility. Among the known mitoepigenetic mechanisms, mitochondrial microRNAs (mitomiRs) have attracted substantial attention due to their potential roles in modulating mitochondrial gene expression in response to environmental stressors. Since many problems with male fertility are known to result from environmental factors, there has been increasing interest in studying epigenetic mechanisms that contribute to male reproductive function. This review explores the current literature regarding different mitoepigenetic mechanisms and their implications for male mammalian fertility, focusing primarily on the known and potential involvement of mitomiRs in model species and humans. Understanding mitoepigenetics may contribute to the development of non-invasive diagnostic biomarkers and individualized therapeutic approaches to male infertility due to their stability in body fluids, tissue specificity, and sensitivity to disease states.

## 1. Introduction

Male mammalian fertility comprises a complex series of traits influenced by anatomical, genetic, hormonal, and environmental variables [1]. In clinical settings, semen quality is measured by parameters such as sperm concentration, motility, morphology, membrane integrity, and mitochondrial activity [2,3]. Although these are the most commonly employed metrics of male fertility potential, semen quality alone does not always accurately predict fertilization success and subsequent embryonic viability [4]. More specifically, standard assessments do not identify subtle molecular alterations that impair sperm function [5,6]. Among these, epigenetic changes are particularly notable as they cause marked changes in gene expression, are often environmentally induced and reversible but do not cause DNA sequence changes which can be readily identified through sequencing or other genetic approaches. One area of epigenetics that is promising in the context of fertility is known as mitoepigenetics, which refers to epigenetic modifications of the mitochondrial genome and transcriptome. Mitoepigenetic changes, including mtDNA methylation and small non-coding RNA (also known as mitomiR) expression, may represent an important link in the complex relationship between environmental stress, mitochondrial function, and spermatogenesis.

Many recognized limitations of semen analysis in the identification male fertility potential and its underlying causes have led to increasing interest in identifying molecular biomarkers that more fully reflect reproductive health at the molecular level [5]. Genetic mutations and epigenetic alterations are currently being explored as biomarkers that correlate with the functional status of the germline as well as its responsiveness to environmental conditions [5]. Both epigenetic and mitoepigenetic modifications are promising candidate biomarkers for male fertility due to their functional roles in controlling the expression of gene genes that lie at the heart of fertility and the relative ease with which they can be measured. This review examines current literature surrounding mitoepigenetics, focusing on mitomiRs, and places it in the context of male mammalian fertility, along with potential future clinical applications.

## 2. Spermatogenesis

Spermatogenesis is a dynamic process in which diploid spermatogonial stem cells (SSCs) differentiate into mature, haploid spermatozoa [7]. This occurs within the seminiferous tubules of the mammalian testes and can be divided into four major stages: spermatocytogenesis, spermatidogenesis, spermiogenesis, and spermiation [8]. During the initial phase, type A dark (Ad) spermatogonia—the self-renewing stem cell population—give rise to type A pale (Ap) spermatogonia. These differentiate into type B spermatogonia which eventually become primary spermatocytes [7].

As described in Amaral et al., 2013 [9], primary spermatocytes enter meiosis I, resulting in haploid, secondary spermatocytes, which then undergo meiosis II during spermatidogenesis. This produces spermatids. Next, during spermiogenesis, round spermatids become elongated, motile spermatozoa with condensed nuclei and flagella containing tightly coiled mitochondria. Finally, during spermiation, mature spermatozoa are released into the lumen of the seminiferous tubules, after which they move to the epididymis. The capacity to fertilize an oocyte is acquired during epididymal transport. Disruptions in cellular signaling, genomic integrity, metabolic function, or gene expression during spermatogenesis are likely to negatively impact sperm quality and fertility outcomes [10]. The impact of mitochondrial dysfunction on male fertility has been very recently reviewed [11].

## 3. Environmental Stress and Male Fertility

Living organisms are exposed to, and influenced by, an extraordinary range of environmental stressors during their lifetimes. The responses to these insults are often assumed to be associated with genetic diversity, where subtle inherited or induced differences in the coding sequence of fertility-associated genes affect the organismal response [12]. However, epigenetic changes in response to environmental cues, particularly during development, may also be mediators of long-term fertility changes and other health effects [13]. Common environmental stressors include temperature fluctuations, chemical exposure, nutritional deficiencies, and oxidative stress, all of which have the potential to impair spermatogenesis [14]. One of the most common environmental stressors is heat stress; temperature plays a critical role in male mammalian reproductive performance [15]. Consequences of heat stress include testicular cell atrophy, reduced sperm count, and temporary infertility [16]. Previous studies have illustrated the association between poor fertility parameters and increased scrotal subcutaneous temperature, characterized by testicular weight loss, a period of infertility, and a gradual return to baseline fertility over one or two spermatogenic cycles [16]. Upon exposure, cells express heat-shock proteins (HSPs) which are molecular chaperones that function to maintain proteins in conformations that retain normal function [17]. HSP expression is partly regulated by epigenetic mechanisms like microRNAs, DNA methylation, and histone modifications [17,18]. Abnormal HSP expression due to the activity of these epigenetic mediators is associated with disease states such as cancer [18]. This presents a paradox whereby HSP expression is altered by mechanisms that are caused by the very stressors that HSPs are employed to mediate.

Another important environmental stressor is oxidative stress, which arises due to an imbalance between free radical generation and antioxidant levels [15]. Oxidative stress due to high levels of ROS is conventionally associated with detrimental health outcomes. Spermatozoa are particularly vulnerable to oxidative stress due to their high content of polyunsaturated fatty acids [19,20]. This is compounded by the limited amount of cytoplasm and antioxidant enzymes within sperm, leading to a diminished capacity to combat ROS [20]. This vulnerability is further exacerbated by the high likelihood that abnormal spermatozoa generate large amounts of ROS [20,21]. This relationship is bi-directional, as increased ROS impairs sperm motility through lipid peroxidation, and compromises mitochondrial function, all of which decrease fertilization potential [15,20]. Additionally, oxidative stress induces DNA fragmentation in both the nuclear and mitochondrial genomes [22,23,24]. Interestingly, while high levels of oxidative stress in spermatozoa prevent fertilization, lower levels permit fertilization but have been associated with increased DNA damage [25]. Attempts to repair this damage by the developing zygote have been postulated to result in mutations associated with pregnancy loss and pathologies including childhood cancers [25].

The most common source of ROS in somatic cells is electron leakage from the ETC during cellular respiration [26]. Mitochondrial ROS production at a level that overwhelms the antioxidant defense mechanisms has been associated with several disease states [20,21]. Various forms of cellular ROS exist, however hydrogen peroxide (H_2_O_2_) is particularly damaging due to relatively persistent action and its ability to readily traverse biological membranes [19].

## 4. Mitochondria: Structure, Function, and Dynamics

Mitochondria play numerous roles in the cell including redox signaling, apoptosis, calcium homeostasis, metabolic intermediate biosynthesis, innate immunity, and energy metabolism [6,9,27]. All of these functions are indispensable for normal spermatogenesis [11]. These organelles contain an outer and inner membrane (OMM and IMM, respectively) which establish the inter-membrane space and mitochondrial matrix. Inner membrane invaginations contain specialized structures (cristae) which are essential for energy production [9]. This energy is used in various cellular processes such as the Krebs cycle, oxidative decarboxylation of alpha ketoacids, beta oxidation of fatty acids, and amino acid catabolism [9,27]. In addition, each of these pathways provide reducing equivalents (NADH2 and FADH2) which are required for oxidative phosphorylation (OXPHOS) [27]. Within the IMM, 5 multi-enzymatic complexes comprising the electron transport chain (ETC) and ATP synthase, are employed during OXPHOS [27,28]. Electron transfer in combination with proton movement from the matrix to the intermembranous space results in a mitochondrial membrane potential (MMP). Changes in this membrane potential can be indicative of mitochondrial dysfunction or excessive endogenous ROS.

### 4.1. Mitochondrial Fusion and Fission

Mitochondria are morphologically, numerically, and functionally dynamic, adapting rapidly to cellular demands and stress [29]. To effect these changes, they utilize highly regulated processes like fusion and fission to alter their number and functional characteristics in order to maintain normal cellular function and energy metabolism [30].

Mitochondrial fusion occurs when two mitochondria merge into a single, continuous organelle. This process mixes mitochondrial contents which in turn can dilute damaged components and promote functional complementation between mitochondria [16]. Fusion also helps maintain mitochondrial bioenergetics under stressful conditions by preserving MMP and supporting ETC function [31,32].

The process of fusion actually encompasses two distinct events due primarily to the presence of the double mitochondrial membrane [31]. These processes are mediated by three, large GTPase proteins: optic atrophy protein 1 (OPA1) is responsible for inner membrane fusion, and Mitofusin 1 and 2 (MFN 1 and 2) which facilitate outer membrane fusion [33,34]. Proper fusion promotes cell survival and metabolic health, while preventing mitochondrial heterogeneity [35]. This is highlighted by the link between MFN2 dysfunction and various neurodegenerative diseases and metabolic disorders [33]. This link is also observed in reproduction, as deletions of either MFN protein has been linked to DNA oxidation and apoptosis in differentiating male germ cells, ultimately leading to infertility [32].

Conversely, mitochondrial fission entails the division of one mitochondrion into two. This enables mitochondrial proliferation, redistribution during cell division, and segregation of damaged regions for degradation [36]. This process is crucial during mitosis, apoptosis, and cellular responses to metabolic stress [37].

Mitochondrial fission is largely driven by cytosolic GTPase dynamin-related protein 1 (DRP1) [38]. DRP1 travels to the OMM and forms physical rings around the mitochondrion. These rings constrict the organelle, ultimately separating it into two distinct organelles [39]. The process is typically coordinated and localized to sites where the endoplasmic reticulum (ER) contact the mitochondria, facilitating accurate positioning and division [40]. Dysregulated fission has been implicated in mitochondrial dysfunction, ROS accumulation, and apoptosis [41]. In terms of male reproduction, any of these consequences may impair spermatogenesis and sperm function [31].

### 4.2. Mitophagy–A Form of Autophagy

One critical element of quality control during cell division involves the elimination of dysfunctional and/or redundant mitochondria. This is carried out through the process of mitophagy (a selective form of autophagy) [31]. The process begins with the recruitment of microtubule-associated protein 1A/1B light chain 3 (LC3) to the autophagosomal membrane [31]. Here, it binds to the mitochondria which, on the OMM, expresses mitophagy receptors [31]. This effectively “tags” the damaged mitochondria as autophagosome “cargo”. The newly formed mitophagosomes subsequently fuse with lysosomes for degradation [31]. When mitophagy fails, damaged mitochondria generate excessive ROS and increased levels of pro-apoptotic factors, leading to cellular death. In the context of male germline cells, mitophagy removes defective mitochondria during spermatogenesis and may influence the selection of sperm with optimal mitochondrial function [42].

### 4.3. Mitochondria and Apoptosis

Mitochondria also serve as key regulators of the intrinsic apoptosis pathway which is initiated by internal cellular stress such as DNA damage, oxidative stress, or developmental signals. The mitochondrial outer membrane becomes permeabilized by pro-apoptotic members of the BCL-2 family (e.g., BAX and BAK), leading to the release of cytochrome c into the cytosol [43]. This activates caspase-9 and other downstream “effector” caspases, leading to cell death [44].

Mitochondrial-mediated apoptosis is critical for the elimination of defective germ cells and for overall testicular homeostasis [11]. However, inappropriate activation of this pathway leads to excessive germ cell loss and reduced sperm count or quality [11]. Therefore, a tightly regulated balance between pro- and anti-apoptotic processes is necessary for the maintenance of male fertility.

### 4.4. Mitochondrial Ferroptosis

One additional category of regulated cell death that is dependent on mitochondrial activity is called ferroptosis. This process is characterized by iron-dependent lipid peroxidation, making it distinct from apoptosis, necrosis, and autophagy. It has been demonstrated that, in certain cellular contexts, mitochondria play a central role in this process by generating sufficient lipid ROS to initiate ferroptosis [45]. Mechanistically, the mitochondrial electron transport chain (ETC) and tricarboxylic acid (TCA) cycle contribute to ROS accumulation, which, in the presence of iron, promotes peroxidation of polyunsaturated fatty acids within mitochondrial and cellular membranes. Ultimately, this disrupts membrane integrity, triggering cell death [46].

Recent evidence suggests that the process of ferroptosis is important in some instances of testis pathophysiology [47]. Iron metabolism in the testis is tightly controlled, as the maintenance of spermatogenesis and testosterone synthesis are both iron-dependent [47]. Specifically, peritubular myoid cells, Leydig cells and Sertoli cells store the bulk of cytoplasmic ferritin in the testes [47]. During the process of spermatogenesis, spermatogonia release iron, transferring it to round spermatids [47]. These in turn transfer the iron to Sertoli cells, where the iron is stored by ferritin which circulates to the primary spermatocytes [47]. Finally, the last of the iron is taken up by elongating spermatids [47].

In excess, cellular iron overload induces oxidative stress and lipid peroxidation, affecting overall sperm quality [47]. This is consistent with observations that abnormal iron levels are present in the semen of patients with some cases of asthenospermia [47]. In further support of roles for ferroptosis in male fertility, iron overload and sperm defects due to testicular injury can be reversed after treatment with deferoxamine (an iron chelator) [47]. Together, these findings strongly support a potential link between ferroptosis and male reproductive dysfunction and reveal a promising potential strategy for treating infertility [47].

### 4.5. Vulnerability of the Mitochondrial Genome

Mitochondria are maternally inherited and contain their own highly compact, circular genome (mtDNA), which encodes 13 OXPHOS subunits, 22 transfer RNAs (tRNAs), and 2 ribosomal RNAs (rRNAs) [48,49]. Unlike nuclear DNA, mtDNA lacks histones and is instead packaged into nucleoprotein structures, called nucleoids. Nucleoids contain either single (monomeric) or multiple (multimeric) mtDNA copies depending on cell type [50,51,52]. Nucleoids are formed in large part by transcription factor A, mitochondrial (TFAM), which coats mtDNA and bends it into U-shaped turns at promoter regions, functionally regulating genome accessibility for transcription, replication, and repair [51,52]. Genes within the nuclear genome typically have specific promoters upstream of their transcription start sites. In contrast, the mitochondrial genome contains three principal promoter regions: the light strand promoter (LSP) and two heavy strand promoters (HSP1 and HSP2) [48,53]. Consequently, the mitochondrial genome is transcribed into long, polycistronic transcripts that require extensive processing before acquiring competence for translation [48,53]. Due proximity of mtDNA to the ETC, its limited repair capacity, and its lack of protective histones, it is particularly vulnerable to oxidative stress [54]. Any resultant damage may compromise OXPHOS activity and impair sperm motility [55].

Collectively, the mitochondrial processes described above create a highly regulated, integrated network guiding cellular maintenance, stress responses, and metabolic regulation [31]. In male germ cells, this network maintains mitochondrial quality and functionality, which are critical for the support of the many energy-intensive processes required for normal spermatogenesis and motility. As a result, dysregulation of any process in this network may have substantial consequences for spermatogenesis.

### 4.6. Mitonuclear Communication

The nuclear and mitochondrial genomes are normally in constant communication to coordinate and maintain cellular homeostasis. Specifically, mitochondrial function relies heavily on nuclear-encoded proteins, while nuclear gene expression is simultaneously influenced by signals generated by mitochondrial activity. Signals from the nucleus that affect mitochondrial function and gene expression are considered *anterograde* signaling, whereas *retrograde* signaling involves mitochondrial signals that alter nuclear gene expression [56,57].

Anterograde communication regulates mitochondrial biogenesis, dynamics, and metabolism. Although mitochondria are responsible for a wide range of necessary cellular functions, mtDNA lacks many of the genes to encode proteins for OXPHOS and other biosynthetic functions [58]. As a result, the majority of mitochondrial proteins and enzymes, including those involved in OXPHOS, transcription, translation, and quality control, are encoded by, and transcribed from, the nuclear genome after which they are translated in the cytoplasm, and imported into the mitochondria [58]. For example, nuclear-encoded transcriptional regulators such as nuclear respiratory factors (NRF1 and NRF2) and peroxisome proliferator-activated receptor gamma coactivator 1-alpha (PGC-1α), play central roles in coordinating mitochondrial gene expression. This and other examples of anterograde communication ensure proper assembly of mitochondrial respiratory chain complexes and appropriate cellular adaptation to changing energy demands [59].

Conversely, retrograde communication allows mitochondria to relay signals regarding their functional status to the nucleus, leading to changes in gene expression. This response may involve energetic cues, mitochondrial stressors, mtDNA mutations, or ROS and calcium signaling [56]. Retrograde communication leads to transcriptional reprogramming in the nucleus that promotes mitochondrial adaptation or repair [56]. This process has also been called “mitonuclear feedback” [56]. Key retrograde signals include alterations in calcium homeostasis, ATP/ADP ratios, ROS levels, and the release of mitochondrial metabolites such as α-ketoglutarate, succinate, and acetyl-CoA, which act as cofactors for enzymes that influence chromatin dynamics [56]. These pathways integrate mitochondrial activity with nuclear gene expression, directing bioenergetic responses to cellular stress. Depending on the context and specific mitochondrial stress signal, an ‘integrated stress response’ may be initiated, which triggers a whole-cell response by decreasing overall cell protein synthesis [56].

In reproduction, mitonuclear communication is particularly important during gametogenesis and early embryogenesis, where highly coordinated nuclear and mitochondrial activity ensures proper epigenetic reprogramming, chromatin remodeling, and energy supply for development [58,59]. Male germ cells, in particular, have distinct metabolic requirements at each stage of spermatogenesis [31]. For example, spermatocytes and spermatids rely on lactate and pyruvate, and by extension, mitochondrial OXPHOS for energy [31]. Thus, coordinated mitonuclear signaling during these critical windows of development ensures that mitchondrial metabolism levels are consistent with cellular demands. Therefore, dysregulated communication can disrupt germ cell quality, embryonic competence and, ultimately, fertility outcomes.

### 4.7. Mitochondrial Function in Male Fertility and Infertility

Although recognized as an essential element of spermatozoa function, the specific roles of mitochondria in the sperm is complex and often contradictory in the literature [27,43]. During spermiogenesis, much of the cell cytoplasm as well as some mitochondria are eliminated as residual bodies, before being phagocytosed by the surrounding sustentacular cells [6]. This process leaves the mature gamete nearly devoid of mtDNA [6,9,27]. The remaining mitochondria are highly condensed, more metabolically efficient, and take on a new structural conformation in the spermatozoan midpiece [27]. These later mitochondria are tightly linked to one another in a helical formation within the axoneme known as the mitochondrial sheath [27]. The primary role of this sheath is to produce the ATP necessary for sperm functions with high energy requirements, including motility, capacitation, hyperactivation, acrosome reaction, and oocyte penetration. Consequently, mitochondrial function is tightly linked with sperm motility, one of the key parameters of semen quality [27]. Indeed, numerous studies have highlighted the relationship between mitochondrial function and sperm quality [6,27]. Highlighting this relationship, differences in MMP provide an estimate of overall mitochondrial metabolic function, and MMP is positively correlated with sperm motility and viability [6]. MtDNA integrity is another emerging biomarker of male fertility. Studies have demonstrated that increased mtDNA copy number or the presence of mtDNA deletions in sperm are associated with poor fertilization outcomes, impaired motility, and idiopathic infertility [60]. Specifically, May-Panloup [60] found the mtDNA content in poor quality sperm to be up to 28 times higher than that in normal samples. Since much of the mitochondria/mtDNA content is lost during early spermatogenesis, the inverse relationship between mitochondria/mtDNA content and semen quality may suggest an upstream alteration in sperm development, specifically during spermiogenesis and failure to adequately eliminate mitochondria. It has been suggested that achieving optimal mitochondrial content secures fertilization ability, which further supports this [6]. The reason for this critical reduction in mitochondrial content is unknown. However, one study suggests that reducing the number of mitochondria in turn reduces the likelihood of ROS-mediated DNA damage [6].

Given their integral role in sperm bioenergetics, redox regulation, and apoptosis, mitochondria are clearly critical participants in the physiological and pathological determination of male fertility. Understanding how mitochondrial function is epigenetically regulated during spermatogenesis represents a promising avenue for the discovery of unrecognized infertility mechanisms and therapeutic targets.

## 5. Mechanistic Links Between Environmental Stress and Spermatogenesis

While environmental stress clearly has significant effects on fertility, the underlying molecular mechanisms that link the two are incompletely understood. Both heat and oxidative stress have been shown to alter gene expression, induce germ cell apoptosis, and impair spermatogenesis [61]. Specifically, testicular heat stress results in stage-specific apoptosis in developing germ cells, while oxidative stress is associated with reduced MMP and sperm viability [62,63].

It is increasingly evident that mitochondria are contributors to ROS generation and oxidative stress, while simultaneously acting as targets of oxidative damage [64]. This reciprocal relationship is compounded by the exacerbation of cellular injury resulting from mitochondrial dysfunction, particularly in cells with high mitochondrial energy demands such as spermatocytes and spermatozoa [65]. Despite these observations, the major linking factors between environmental stress and functional molecular and gene expression changes in the mitochondria have not been identified. These gaps in knowledge highlight the need to examine cellular mechanisms of gene expression changes in response to environmental cues. The regulation of gene expression through altered epigenetic processes at the level of the nuclear and mitochondrial genomes represents a promising avenue of research to help understand some important environmental impacts on male fertility.

## 6. Epigenetics: Potential Molecular Mechanisms of Environmental Effects on Spermatogenesis

**Figure 1:**
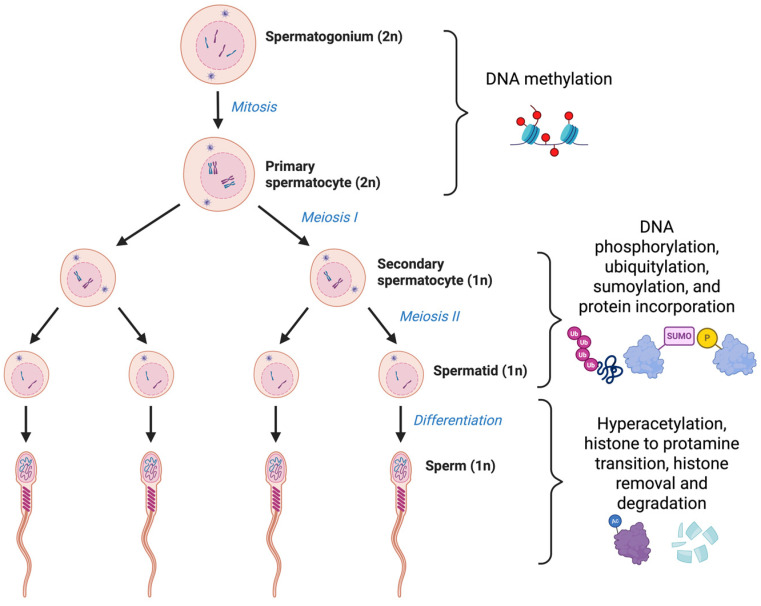
Epigenetic events during spermatogenesis. Epigenetic events begin in mitotic primordial germ cells with DNA methylation, followed by phosphorylation in meiotic primary and secondary spermatocytes. This is followed by ubiquitylation, sumoylation, and protein incorporation (all of which are involved in XY body formation). Finally, hyperacetylation occurs during spermiogenesis to assist in the histone-protamine exchange (Created with BioRender.com).

The field of epigenetics investigates mechanisms that influence gene expression through processes like DNA methylation, histone modifications, and the activity of small non-coding RNAs [66,67]. Epigenetic processes are likely to underlie many of the mechanisms by which environmental stressors modulate gene expression and fertility through their effects on spermatogenesis. Regulation of the epigenome is not only crucial for cell fate determination (i.e., differentiation stages) but also contributes to the overall maintenance of tissue homeostasis throughout the male reproductive system [68]. In the context of male fertility, critical steps in the establishment of the epigenome occur in prospermatogonia during fetal development and multiple epigenetic modulation events support spermatogonial stem cell self-renewal and differentiation during spermatogenesis later in life [68]. (Figure 1).

The concept of biologically embedded environmental influences has gained significant traction since its recognition in the early 1940s [69,70]. Importantly, although these genomic changes are heritable, the DNA sequence itself remains unchanged in most cases [66,67,71,72]. In essence, epigenetic changes alter the phenotype of sperm itself and the resulting progeny without genotypic changes. The epigenetic status of a gamete reflect both genetically programed developmental processes and the animal’s unique environmental history [68]. As previously mentioned, cells undergo two waves of genome-wide epigenetic reprogramming during mammalian development. The first occurs in the zygote, and the second occurs postnatally in primordial germ cells (Figure 1) [68]. This suggests that there are numerous “windows” where the key epigenetic events of gamete development and spermatogenesis can be altered by environmental cues or stressors, changing the epigenetic characteristics of the cell which are sustained for the life of the individual and heritable by their progeny [66,67,71,73]. In this way, both genetic and epigenetic abnormalities can contribute to male mammalian infertility [71]. In support of this, epigenetic alterations in sperm have been strongly correlated with overall sperm quality [71,74]. For example, exposure to heat or oxidative stress has been shown to disrupt DNA methylation patterns, alter the expression of histone-modifying enzymes, and shift non-coding RNA profiles [75,76]. In the germline, these ‘epimutations’ not only alter genome activity, but may increase disease susceptibility of subsequent generations [76].

Although typically employed to overcome reproductive challenges, assisted reproductive technologies have been shown to induce epigenetic alterations in sperm [71]. These alterations may be the result of utilizing sperm that has not fully undergone epigenetic reprogramming, or from undertaking in vitro embryo procedures while epigenetic reprogramming is underway [71]. This highlights the importance and sensitivity of these critical reprogramming events, as well as the potential consequences of manipulating germ cells during epigenome establishment.

## 7. Epigenetic Mechanisms Involving the Nuclear Genome and Transcriptome

The primary epigenetic mechanisms identified to date include DNA methylation, histone modification (methylation, acetylation, phosphorylation, ubiquitination, and sumoylation), as well as small non-coding RNA pathways [66,67,72,74,75,76]. These pathways integrate environmental and other intercellular signals to influence chromatin accessibility, transcriptional activity, stability and translation of transcribed RNAs, ultimately playing essential roles in germ cell development, spermatogenesis, and fertility [67,77,78]. The characteristics and processes of epigenetics involving the nuclear genome in reproduction have been comprehensively reviewed elsewhere [68,79]. This review primarily focuses on mitoepigenetic mechanisms, with an emphasis on mitochondrial-associated small RNAs and their role in fertility.

Small non-coding RNAs such as miRNAs typically alter the stability and translation of mRNAs encoded by the nuclear and mitochondrial genomes [80]. MicroRNAs are evolutionarily conserved, single-stranded, 19–25 base pair processed transcripts that regulate gene expression through their post-transcriptional activity (mRNA degradation or translational repression). miRNAs have several features that support their utility as biomarkers; these include their highly conserved nature, detectability in biological fluids, stability, involvement in pathway regulation, tissue and cell specific expression, and their roles in disease pathophysiology [80].

Several thousand individual miRNAs have been described or predicted, and each has a specific target sequence, allowing them to modulate the expression of a repertoire of genes that is relatively unique for each individual miRNA [81]. The pattern of miRNAs expressed in any tissue is often highly specific with respect to cell type and developmental stage, making them potentially specific biomarker candidates for tissue function and health in the reproductive system and elsewhere [80,82].

MiRNA biogenesis proceeds through one of several pathways [83]. Most are synthesized via the canonical biosynthetic pathway from the nuclear genome. In this pathway, RNA polymerase II transcription produces a primary-miRNA transcript (pri-miRNA) which is cleaved in the nucleus by Drosha, resulting in a shorter pre-miRNA [83]. A complex of transport proteins, composed of exportin 5 and GTP-binding nuclear protein, export the pre-miRNA to the cytoplasm where it is further cleaved by Dicer, forming a double-stranded RNA duplex [83]. After dissociation, one strand of this duplex becomes a mature miRNA which then associates with a multiprotein complex containing an Argonaute (AGO) protein, collectively known as the RNA-induced silencing complex (RISC) [83]. Complementarity between the passenger miRNA and sequences in the 3′UTR of target transcripts direct the RISC complex to the mRNA for translation repression or degradation [83].

In addition to the canonical pathway, miRNAs can also be generated via several non-canonical pathways, which bypass one or more steps of the classical processing machinery. These can be further classified into Drosha-independent and Dicer-independent pathways. One example of a Drosha-independent pathway is the mirtron pathway, in which short intronic sequences (mirtrons) are spliced by the spliceosome to form pre-miRNA-like hairpin structures, circumventing the need for Drosha-dependent processing [84]. Mirtron-derived pre-miRNAs are then exported and processed by Dicer in the cytoplasm, utilizing pathways similar to canonical pre-miRNAs [85]. Other examples of Drosha-independent pathways include small nucleolar RNA (snoRNA)-derived and tRNA-derived pathways in which snoRNAs and tRNAs fold into hairpin structures and enter the biogenesis pathway through Dicer and/or AGO activity [86]. A well-characterized example of miRNA processing by a Dicer-independent pathway is miR-451. The miR-451 pre-miRNA is cleaved directly by an Argonaute 2 (AGO2)-dependent pathway, bypassing Dicer entirely [83]. These alternative pathways broaden the regulatory potential of miRNAs, provide redundancy if the canonical pathway is compromised and can be differentially activated depending on tissue type, developmental stage, or cellular stress [87].

## 8. Mitoepigenetics

### 8.1. Modifications to Mitochondrial DNA

Recent literature has suggested that, similar to nuclear DNA, mtDNA is also subject to epigenetic modification [28], although the mechanisms appear more limited in scope than those in the nuclear genome. Mitoepigenetics is a specialized subset of the field of epigenetics, wherein epigenetic mechanisms alter mitochondrial gene expression and overall mitochondrial activity. Abnormalities and dysfunction in these mechanisms therefore have the potential to lead to dysfunction and disease states. Although the field of mitoepigenetics is relatively new, and still largely unexplored, the presence of methylated mtDNA as well as various noncoding RNAs in the mitochondria have been well documented [26,28]. For example, regulatory elements of mtDNA, specifically the D-loop, have been found to be enriched with meCpG [88]. Methylation at this site can affect the binding affinity of TFAM and is associated with the suppression of gene expression [28]. Furthermore, epigenetic enzymes like DNA methyltransferases (DNMTs), have been found to exist at low concentrations within the mitochondria, suggesting altered mtDNA methylation [89,90]. Additionally, a significant correlation between mtDNA methylation and ETC subunit expression, which is essential for mitochondrial and cellular function, has been described [16]. Another important mitoepigenetic process involves the action of small noncoding RNAs that are targeted to mitochondrial genes or are transcribed within the mitochondria themselves [28]. The presence of miRNAs in the mitochondria, termed ‘mitomiRs’, was first demonstrated in 2011, where it was postulated that these sequences may act on mitochondrial transcripts or become exported to the cytosol to alter nuclear mRNA expression [91]. Various studies have subsequently identified and expanded the list of these localized mitomiRs [92,93,94].

TFAM is a key regulatory element for mtDNA maintenance, replication, and transcription; TFAM expression is dependent on NRF1 and NRF2 [85]. After transport into the mitochondria, TFAM binds to mtDNA, enhances transcription, and organizes mtDNA into nucleoids in order to maintain mitochondrial genome stability [95]. TFAM expression is tightly linked to mitochondrial biogenesis and cellular energy metabolism [96].

Beyond control of TFAM mRNA levels, mitoepigenetic mechanisms directly modulate TFAM activity within the mitochondria. For example, mtDNA methylation influences TFAM’s interaction with the genome [97]. Specifically, the addition of N6-methyladenine marks to mtDNA, catalyzed by mitochondrial methyltransferase METTL4, reduces TFAM’s DNA-binding affinity and impairs nucleoid formation, leading to reduced mtDNA copy number and transcriptional output [97]. These findings support the role of mitoepigenetic regulation in modulating TFAM function and consequently, mitochondrial homeostasis.

It is postulated that mitochondria evolved through bacterial endosymbiosis, resulting in a lack of histones and a compact genome with few non-coding regions in a pattern typical of prokaryotes [98]. This absence of chromatin structure suggests that other (mito)epigenetic mechanisms such as DNA methylation and RNA-based epigenetic control [99] predominate, in addition to regulation via post-translational modifications of TFAM.

### 8.2. MitomiRs–Small Non-Coding RNAs That Target Genes Associated with the Mitochondria

**Figure 2:**
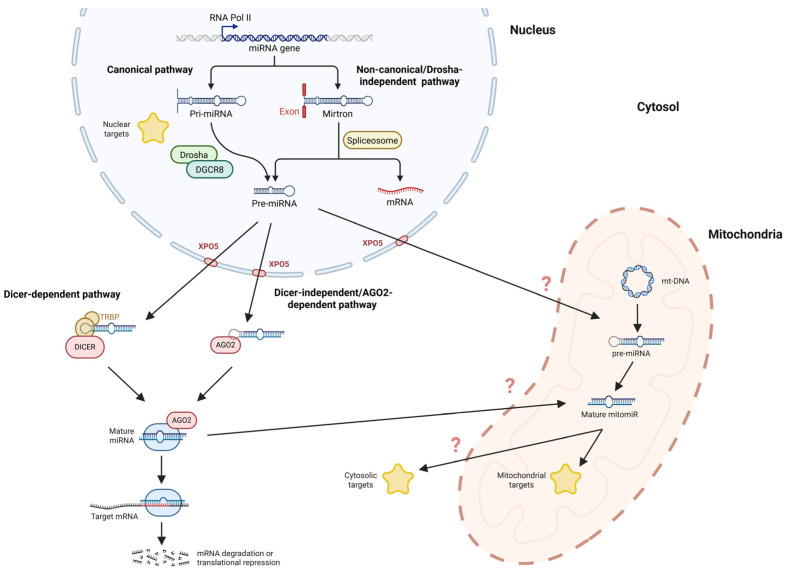
Canonical, non-canonical, and proposed mitomiR biogenesis and transport mechanisms (Created with BioRender.com). In the canonical pathway, RNA polymerase II transcription produces a primary-miRNA transcript which is cleaved in the nucleus by Drosha, resulting in a pre-miRNA. Transport machinery exports the pre-miRNA to the cytoplasm where it is further cleaved by Dicer, forming the double-stranded RNA duplex. One strand of this duplex becomes the mature miRNA which, when associated with RISC, directs Agonaute protein binding to target specific mRNAs for translation repression or degradation. Non-canonical miRNA pathways bypass one or more canonical steps. Multiple origins and transport pathways are likely involved in mitomiR biogenesis.

The principal focus of this review is the epigenetic roles of mitomiRs, which are post-transcriptional regulators of mitochondrial genes encoded by both mtDNA and nuclear DNA [91,94]. MitomiRs modulate mitochondrial gene expression (from the mitochondrial genome) through targeting of mitochondrial mRNAs, while also targeting nuclear-encoded mRNAs and their targets [100]. MitomiRs are functionally divided into 3 classes based on their origins and target genes. Nuc-miRs originate in the nuclear genome, are processed in the cytoplasm, and target genes transcribed from the nuclear genome that regulate mitochondrial function [101]. Nuc-mitomiRs are also transcribed in the nucleus but translocate into mitochondria targeting transcripts from mitochondrial genes [101]. Finally, mt-mitomiRs, are transcribed from the mitochondrial genome and act there, targeting mitochondrial genes [101]. A graphical representation of mitomiR biology is presented in Figure 2.

The site(s) and processing steps involved in the maturation of mtDNA-encoded mitomiRs remain unresolved. Several canonical miRNA processing proteins such as Dicer and AGO2 have been detected within mitochondria, suggesting local miRNA processing [102]. For example, AGO2 has been consistently localized to the mitochondrial matrix and outer membrane in multiple cell types, where it may participate in mitochondrial RISC function [65]. Similarly, PNPT1 (also known as PNPase), an RNA import factor known for transporting small RNAs across mitochondrial membranes, has been associated with the presence of nuclear-encoded mitomiRs in the mitochondria [103]. The transport mechanisms that mediate import/export to the mitochondria, and the subcellular location where these processes occur remain unknown. These major issues are extensively discussed in an excellent recent review [104].

Dicer protein localization to mitochondria remains controversial. Some studies have reported mitochondrial Dicer activity, supporting the hypothesis that precursor miRNAs (e.g., pre-miRNAs) are processed in situ [91], while others have failed to detect Dicer in highly purified mitochondrial fractions, raising the possibility that mitomiR processing likely occurs in the cytoplasm prior to mitochondrial import [102]. This ambiguity is complicated by the challenge of isolating pure mitochondrial preparations free from cytoplasmic proteins [94].

These issues are particularly relevant to mitomiR function because the site of miRNA processing may strongly influence their regulatory activities within the organelle. Determining whether mitochondria possess autonomous mitomiR processing machinery or rely entirely on nuclear/cytoplasmic processing should help clarify our understanding of mitonuclear communication and the adaptive capacity of germ cells to modulate mitochondrial activity in response to environmental cues.

### 8.3. MtDNA-Encoded mitomiRs

Among the three mitomiR categories described above, mtDNA-encoded mitomiRs are the most debated and due to their potentially unique functions in mitochondrial biology. These small RNAs are hypothesized to originate directly from the mitochondrial genome, offering the possibility of an autonomous regulatory unit within the organelle itself. Early sequencing efforts have revealed small RNA species that map to mitochondrial loci and decrease in abundance after mtDNA depletion suggesting mitochondrial transcription as their source [94]. At the very least, these findings suggest that mitochondria are capable of generating miRNA-like molecules that engage canonical RNA-silencing machinery.

Functional assays have further supported the idea that mt-mitomiRs participate in both local and systemic gene regulation. Some candidates have been postulated to participate in mitochondrial biogenesis through the modulation of TFAM and PPARGC1A gene expression and mtDNA copy number [94]. PPARGC1A encodes PGC-1α which, as previously mentioned, is crucial for mitochondrial biogenesis [105]. These results suggest that mt-mitomiRs may contribute to mitonuclear communication by altering mitochondrial transcripts and by coordinating adaptive responses that extend beyond the organelle.

The study of mitoepigenetics remains a challenging field to pursue for several technical reasons. Distinguishing mtDNA-derived small RNAs from sequencing artefacts or reads misaligned to nuclear-derived DNA segments is difficult, and this is compounded by the relatively low abundance of mt-mitomiRs isolated during sample preparation [106,107]. In addition, mitochondrial isolation experiments risk cytoplasmic contamination, confounding the interpretation of co-localization with proteins like AGO2. These technical obstacles highlight the strict requirement for multiple, distinct, and independent validation strategies to definitively establish mitochondrial origin for mitoepigentic regulatory candidates. Examples of methods used to validate integrity, quality, and purity of mitochondrial RNA include the use of Qubit Fluorometers and automated electrophoresis methods [106].

If the presence and functional roles of mt-mitomiRs can be more clearly established and characterized, an additional layer of mitochondria-dependent regulation of gene expression and cellular homeostasis is likely to emerge. This would broaden the mitochondria’s current status as a target of nuclear-encoded miRNA activity and expand again the regulatory possibilities for mitochondria to both influence and be influenced by cellular, intercellular and environmental cues.

## 9. Environmental Impacts on Mitoepigenetic Mechanisms and Male Fertility

Because mitochondria lie at the center of cellular metabolic activity, ROS generation, apoptosis regulation and signaling, it is understandable that they have evolved as important mediators of responses to environmental changes [80]. Environmental conditions such as heat and oxidative stress directly influence mitochondrial activity and many of these effects occur through epigenetic and mitoepigenetic changes including altered mitomiR expression [16,28]. The ability to sense, integrate, and transduce information regarding environmental changes optimizes the balance between energy efficiency and the risk of cellular damage [108].

ROS are primarily generated by mitochondrial OXPHOS and play complex roles in both the regulation and disruption of cellular homeostasis [97]. While physiological levels of ROS are essential for cellular signaling, excessive production overwhelms antioxidant defenses and damages cellular lipids, proteins, and nucleic acids [20,21]. The lack of protective histones and limited repair capacity of mtDNA renders mitochondrial DNA particularly susceptible to damage in the presence of ROS [6,27]. In sperm, this decline results in permanent damage to the motility apparatus, reducing fertilization capacity and overall fertility [6,27].

Changes in mitomiR expression under stressful conditions represents an important potential layer of regulation in response to environmental cues that may alter ROS generation and cellular metabolism [99]. For example, miR-210 targets mitochondria electron transport chain and TCA cycle factors, ultimately altering energy metabolism and redox balance [109]. Within the context of male mammalian reproduction, shifts in mitomiR profiles have been observed following environmental stress, altering mitochondrial transcripts and disrupting energy metabolism within germ cells [80]. From the perspective of male reproductive activity, these findings suggest that mitomiRs are likely to play an active role in modulating mitochondrial responses to environmental changes, ultimately affecting spermatogenesis and fertility.

## 10. MitomiRs and Mitoepigenetic Influences on Mitochondrial Function and Dynamics

Once considered autonomous and relatively static organelles primarily responsible for energy metabolism, mitochondria are now recognized to be highly integrated participants in complex intracellular communication networks. Ongoing changes in their morphology and activity help safeguard normal cellular function [30]. Mitoepigenetic mechanisms, particularly mitomiRs, regulate these through their effects on processes such as apoptosis, fusion, fission, ferroptosis, mitophagy, and autophagy. Because mitochondria often act as environmental sensors, mitomiRs likely serve as dynamic epigenetic mediators that bridge external and internal cues with functional mitochondrial changes [108].

Mitochondrial number and morphology, governed by the balance between fission and fusion, are subject to mitomiR dependent regulation. For example, miR-324-5p suppresses mitochondrial fission by targeting mitochondrial fission regulator 1 (Mtfr1) [110]. Interestingly, miR-324-5p is involved in intracellular signal transduction affecting spermatogenesis, the acrosome reaction, and testicular function [111]. Additionally, it has been shown to protect against oxidative-stress induced apoptosis in endothelial progenitor cells [112]. MiR-140 and miR-106b modulate mitochondrial morphology and function by regulating mitofusin 1 and 2 (MFN1/2), and the fission protein dynamin-related protein 1 (DRP1) [113]. MiR-140-3p has been found to be associated with the proliferation and apoptosis of Sertoli cells [114], and miR-106b is critical for testicular homeostasis and male fertility [115]. Similar to miR-324-5p, miR-106b has also been shown to protect against oxidative stressed-induced mitophagy [116].

Apoptosis, mitophagy and autophagy, processes that remove damaged or dysfunctional mitochondria, are also influenced by mitoepigenetic regulation. For example, specific mitomiRs have been implicated in modulating mitochondrial apoptosis by targeting the B-cell lymphoma-2 (BCL-2) family of proteins. Specifically, miR-181a, miR-34a, and miR-146a suppress the anti-apoptotic transcript encoding BCL-2, facilitating cytochrome c release and caspase activation [117]. Mir-181a, miR-34a are key regulators of spermatogenesis [118,119], and miR-146a is highly regulated during spermatogonial differentiation [120].

MiR-762 is an another mitomiR that has been linked to increased apoptosis in myocardial cells by binding to the coding sequence of ND2, resulting in reduced ATP production, elevated ROS, and cell death [121]. In mammalian-reproduction, miR-762 promotes immature Sertoli cell growth [122]. Similarly, miR-214 is encoded in the nucleus, but translocates into the mitochondria where it targets mitochondrial ND6 and ND4L [123]. Apoptosis is impeded when miR-214 is inhibited [123]. One study found that miR-214 regulates spermatogonium apoptosis and aging [124], and another highlighted its protective effects in erythroid cells undergoing oxidative stress [125]. In hepatocellular carcinoma cell lines, miR-518d-5p targets p53 Upregulated Modulator of Apoptosis (PUMA), which is a pro-apoptotic mitochondrial gene [126]. Interestingly, miR-518d is differentially expressed in spermatozoa from infertile men [127]. Additional miRNAs known to regulate autophagy-related genes may participate in the mitoepigenetic modulation of mitophagy, thus maintaining sperm mitochondrial health by selectively clearing damaged mitochondria [97].

With regard to mitomiR roles in other important mitochondrial processes, mitomiR-3 has been identified as a regulator of ferroptosis [128]; inhibition leads to metabolic reprogramming towards a pro-ferroptotic phenotype, with increased iron accumulation and lipid peroxidation and elevated polyunsaturated fatty acid metabolite levels [120]. This is accompanied by the suppression of glutathione peroxidase 4 (GPX4), a critical enzyme in the defense against ferroptosis [128]. ZEB1 (Zinc Finger E-Box Binding Homeobox 1) directly inhibits GPX4 transcription and has also been found to be targeted by 11 different mitomiRs [128]. While no published studies have investigated the role of mitomiR-3 in male reproductive cells, the emerging role of this and other mitomiRs in regulating ferroptosis has several implications for male fertility. Dysregulation would be predicted to lead to ferroptotic cell death, poor sperm quality and decreased fertility.

Together, these observations in different cell types suggest that mitomiRs integrate environmental and cellular stress signals to epigenetically modulate mitochondrial function, dynamics, and quality control. Because cellular energy, ROS homeostasis, apoptosis, and sperm motility are all tightly linked to mitochondrial integrity, any mitomiR-driven alterations in mitochondrial structure or function may significantly impact male mammalian reproductive potential [129]. Therefore, this proposed regulatory pathway would be likely to contribute to the ongoing mitochondrial and cellular changes that occur during spermatogenesis.

## 11. Technical Considerations for Future Investigation

Future investigations into mitochondrial dynamics in spermatogonia should include the study of *mitoepigenomics* which takes a multi-omics approach to mitochondrial genomic regulation [130]. Investigating the mitochondrial epigenome, transcriptome, proteome, metabolome, and interactome paint a broader picture of mitochondrial regulation [130]. This may allow researchers to tease out some of the remaining questions regarding mitochondrially encoded mitomiR biogenesis and transport, and the interaction of different mitoepigenetic pathways due to coinciding environmental stressors. Approaches like targeted bisulfite sequencing [131], single-cell sequencing [132], and integrating multi-omics datasets [133] have recently unveiled dynamic changes and interactions concerning mitochondrial genomic regulation.

## 12. Conclusions: Biomarkers, Therapeutic Potential

Studies investigating the epigenetic regulation of gene expression, tissue function and health have dramatically altered our understanding of genome-environment interactions. Mitoepigenetics, a subdiscipline within epigenetics that focusses on processes affecting mitochondria, is relatively newer but is also likely to provide substantial insight into how cells respond to environmental cues. Several avenues remain unexplored such as the exact mechanisms of mitomiR transport into and/or processing within the mitochondria, as well as the interactions of various mitoepigenetic processes within the same cell. For male reproduction, mitoepigenetics offers several new perspectives coupling well-recognized roles for this important organelle in sperm development and function. This is particularly true for small RNAs such as mitomiRs which are increasingly recognized to couple mitochondrial and cellular function to environmental changes.

Mitochondria are slowly being recategorized as transducers of biological information, significantly expanding their classical role as “powerhouses” for the cell. Mitoepigenetic pathways respond to the environment and influence mitochondrial processes such apoptosis, fusion, fission, ferroptosis, autophagy, and mitophagy. Consequently, disruptions in these pathways by environmental or other insults during spermatogenesis are likely to contribute to altered sperm characteristics and quality, contributing to infertility in some cases.

In addition to improving our mechanistic understanding of how environmental factors impact mitochondrial function and downstream effects such as fertility, the field of mitoepigenetics presents several potential opportunities for clinical assessments and interventions. Since mitoepigenetic changes are likely to impact fertility, direct assessment of the “mitoepigenetic state” of sperm or testis may reveal fertility issues that traditional semen parameters do not. Because mitochondria integrate multiple environmental signals, such evaluations may reflect multiple, widespread environmental impacts on the process of spermatogenesis in sensitive and subtle ways. If changes in cellular mitomiR levels are maintained through the stages of spermatogenic differentiation, their patterns of expression may represent useful non-invasive biomarkers of sperm mitochondrial health. Because they are stable, readily detectable in bodily fluids, and both drive and reflect subtle perturbations in mitochondrial function, their evaluation has the potential to enhance early detection of subfertility and guide subsequent treatment strategies. Furthermore, given their active roles in modulating mitochondrial function and health, therapies that alter specific mitomiR levels could help restore mitochondrial function and improve reproductive outcomes. This personalized approach aligns with broader trends in precision medicine, where molecular profiling guides individualized care.

## Data Availability

No new data were created or analyzed in this study.

## Abbreviations

The following abbreviations are used in this manuscript:

mitomiR	Mitochondrial miRNA
mtDNA	Mitochondrial DNA
ROS	Reactive oxygen species
OXPHOS	Oxidative phosphorylation
MMP	Mitochondrial membrane potential
ETC	Electron transport chain
TFAM	Mitochondrial transcription factor A
